# Evidence for the existence of the A2A-A1 heteroreceptor complex in the rat brain, and comparison of its distribution to that of the A2A-A2A homoreceptor complex

**DOI:** 10.1186/2193-1801-4-S1-P31

**Published:** 2015-06-12

**Authors:** Julia Oflijan, Dasiel Borroto-Escuela, Miles Woolfenden, Michael Di Palma, Luca Pinton, Ismel Brito, Manuel Narváez, Fidel Corales, Antonio Jimenez-Beristein, Luigi F Agnati, Kjell Fuxe

**Affiliations:** Karolinska Institutet, Sweden; University of Tartu, Estonia

**Keywords:** science, microscope, histology

Adenosine receptors play critical roles in cellular processes and signaling and have been shown to form heteromers with diverse biochemical and/or pharmacological activities that are different from those of the corresponding homomers1. However, despite extensive experimental results supporting the formation of adenosine heteromers in heterologous systems, the existence of such heteroreceptor complexes in the brain remains largely unknown, mainly because of the lack of appropriate methodology. Also no systematic study was carried out on heteromers form by adenosine receptor subtypes alone. In this study, we used several experimental approaches2 to investigate whether adenosine receptor A2A and A1 subtypes can form heteromers among themselves. In situ PLA clearly demonstrated that adenosine receptors (A2A-A2A, A2A-A1) exist as homo/heteroreceptor complexes in rat brain. In the hippocampus, A2A-A1 heteroreceptor complexes are mainly localized to the pyramidal cell layer of the Ammon´s horn and the hilo (PoDG). The complex was also observed throughout the piriformis layer. Several distinct differences were apparent between the distribution of the A2A-A1 heteroreceptor complexes and that of the A2A-A2A homoreceptor complex, which could have important functional consequences. Furthermore, bioluminescence resonance energy transfer analysis of adenosine A2A receptors established that they can physically interact in HEK293T27 cells, as both homomers and heteromers. In addition, static/non-dynamical human GPCR data derived from this and other interaction studies were integrated in a large scale graph, called the GPCR heterodimer network (http://www.iiia.csic.es/~ismel/GPCR-Nets/index.html), which provides global insight into adenosine heteromer connectivity, topology and organization in the context of the adenosine receptor subfamily and the GPCR network as a whole.

## References

[1] Borroto-Escuela DO, Romero-Fernandez W, Tarakanov AO, Ciruela F, Agnati LF, Fuxe K (2011). J Mol Biol.

